# Stress and suicide risk among adolescents: the role of problematic internet use, gaming disorder and emotional regulation

**DOI:** 10.1186/s12889-024-17860-z

**Published:** 2024-01-30

**Authors:** Andrés Chamarro, Adrian Díaz-Moreno, Ivan Bonilla, Ramon Cladellas, Mark D. Griffiths, Maria José Gómez-Romero, Joaquín T. Limonero

**Affiliations:** 1 Stress & Health Research Group. Faculty of Psychology, Autonomous University of Barcelona, 08193 Bellaterra (Cerdanyola del Vallès), Barcelona, Spain; 2 International Gaming Unit, Nottingham Trent University, Nottingham, England; 3 Psychology Unit, Egarsat, Mutua Colaboradora con la Seguridad Social nº 276, Barcelona, Spain

**Keywords:** Suicide risk, Problematic internet use, Gaming disorder, Online social networking, Stress, Emotional regulation

## Abstract

**Background:**

Previous studies have associated videogame playing and social media use with suicidal behaviors together with lower stress coping or poor emotion regulation strategies. Due to the inconclusive evidence regarding the factors associated with suicidal behavior, the present study aimed to overcome the limitations of previous research and explored the relationship between adolescent stress, problematic internet use (PIU), gaming disorder (GD), and emotional regulation (ER) in a cross-section design. It was hypothesized that stress would have a direct effect on suicide risk (SR) as well as being mediated by PIU, GD, and ER.

**Methods:**

The participants comprised 430 adolescents (58.4% male) aged between 16 and 19 years. They completed an online survey including the Mobile-Related Experiences Questionnaire, Internet Gaming Disorder Scale-Short Form, Meta-Mood Trait Repair Scale, and Spanish version of the Suicidal Behaviors Questionnaire.

**Results:**

A total of 34.2% of the adolescents (*N* = 147) were at risk for SR. Results also indicated that 30,7% had experienced suicidal ideation at some point in their life, 12.1% had at least one plan to die by suicide, and 5.1% had attempted suicide. Results of path analysis confirmed that stress appeared to be a risk factor for suicide, but that its effects were not mediated by PIU. However, ER and GD mediated the effect of stress on SR. The results suggest that stress is a main risk factor for suicide, especially among adolescents with poor emotional regulation or problematic gaming.

**Conclusions:**

Considering the prevalence of suicide among adolescents, the results of the present study suggest that suicide prevention programs should include emotional regulation strategies, stress coping, and videogaming management skills in the early stages of high school. Providing these protective resources to adolescents will help them face the stressful and changing situations typical of adolescence and will help them to attain greater well-being and satisfaction with life.

## Highlights

34.2% of the adolescents in the sample were at risk for suicidal behavior.

Of the many factors affecting suicide risk among adolescents, problematic use of internet, gaming disorder and difficulties in emotion regulation may play an important role.

Stress is a key factor for suicide risk, especially among adolescents with poor emotion regulation and gaming disorder.

Suicide prevention programs should include stress coping strategies, as well as emotional and videogaming management skills in early stages of high school.

## Introduction

The use of smartphones and videogames is constantly increasing all over the world. In Spain (where the present study was carried out), according to the Spanish Institute of Statistics [1], 95.5% of households have internet access, and 99.5% have mobile phones. The use of online social media platforms is particularly attractive for young people, who represent the age group that has integrated these technologies into their entertainment, education, leisure, and communication to the greatest extent [2]. At the same time, an increase of weekly gaming hours has been observed in Spain, from five hours in 2013 to 7.5 h in 2020 [3].

Consequently, because of the growth of internet use via all kinds of electronic devices with a screen, problematic internet use (PIU), online social networking, and gaming disorder (GD) have all increased in Spain [4]. PIU is an umbrella term to describe the problematic use of the internet, and GD is one of its manifestations [5, 6], and has been included in both the eleventh revision of the *International Classification of Diseases* (ICD-11) [7] and the fifth edition of the *Diagnostic and Statistical Manual* (DSM-5) [8].

Previous studies have associated problematic videogame playing (see [9], for a review) and social media use [2, 10–12] with suicidal behaviors. Also, other negative effects, such as stress [13] or difficulties with emotion regulation, [14] may be associated. Due to inconclusive research exploring the relationship between these factors and suicidal behavior, the present study investigated the relationship between suicidal behaviors and stress, PIU, GD, and ER.

## Suicide

During the coronavirus disease-2019 (COVID-19) pandemic, social and family isolation, together with collective concerns about the economy, physical health, and mental health experienced caused some individuals to experience negative emotions [15, 16]. For young people, this represented an opportunity to increase their use of smartphones and computer devices [17–21]. These factors have facilitated many downstream effects in the mental health of the global population. For example, anxiety and depression rose sharply [22–24], also generating an increase in suicides [25, 26].

Suicide is the main cause of non-natural death in Spain [1] and the fourth leading cause of death in young people between 15 and 19 years of age worldwide [27]. Other manifestations of suicidal behavior should be considered, such as suicide ideation, suicide planning, and suicide attempts [28]. The prevalence of suicidal ideation among adolescents in Spain is between 4.4% and 7.8%, and between 1.5% and 1.8% for suicide attempts [29]. According to the Spanish Foundation for the Prevention of Suicide [30], compared to 2020 (the year in which there was an increase in suicides in Spain, 7.4% higher than in 2019), in 2021 there was a 1.6% increase (1.8% in men and 1% in women). There are many factors that affect suicide risk (SR) among the adolescent population because it is a complex and multidimensional phenomenon with multiple determining factors [31, 32]. In their review, Fonseca-Pedrero et al. [32] proposed that risk factors may be classified as “internal” (e.g., lack of problem-solving skills, ineffective coping strategies) and “external” (e.g., family, and social problems).

The use of new technologies has been added to the many risk factors [33, 2, 34–36] because their use is associated with isolation [37] and depression [38–39], as well as facilitating interaction with other individuals experiencing suicidal ideation or having access to information regarding suicide and/or self-harm on social media platforms [40,41]. A meta-analysis suggested that internet addiction was related to suicidal behaviors [42]. More recently, research has focused on difficulties regulating emotions [43, 44] and stress [45], regarding the relationship between the use of technology and suicide. Other studies with college students [11] suggest that PIU should be considered a risk factor because it increases suicidal behavior by 3.78 times among women and 5.58 times among men, along with social isolation, subjective distress, and depression.

### Stress

Stress is one of the factors that received the most research attention during the pandemic. When there is an epidemic on such a scale, a large proportion of the population are afraid of becoming infected due to lack of understanding about the virus, which ends up generating stress, depression and/or anxiety [46]. The stress experienced during the COVID-19 pandemic has been associated with an increased risk of PIU [47] because individuals attempt to cope with stress through easily accessible behaviors, including the use of various applications on smartphones and computer devices [48]. This effect is consistent with evidence that stressful events (e.g., natural disasters and public health emergencies) result in increased substance abuse [49] and/or PIU [50] at the population level. This may happen because individuals cope with stress using substances (e.g., self-medication) and/or other maladaptive coping behaviors, such as the use of online social media platforms or the playing of videogames to limit their discomfort [51]. Coping with these situations through the intensive use of technology can lead to its problematic use [51–53], especially if its use is forced [54], for example, by study or work reasons. This has become a typical situation during adolescence, in which adolescents are pressured to achieve good academic results to access higher education opportunities.

Another of the consequences of stressful situations is the appearance of suicidal ideation [55]. Here, experiencing high levels of stress for a long period of time increasing the likelihood of suicidal ideation, and, therefore, increasing the probability of attempting or dying by suicide [28], as happened during the COVID-19 pandemic [25], when experiencing cyberbullying [56] or sexting [57]. However, there is an increasing interest into how stress could be influencing suicidal behavior, and some elements influencing the stress-SR association during adolescence have been proposed. For instance, Ballabera et al. [58] observed that adaptive cognitive emotion regulation strategies were mediators between perceived stress and the risk of suicidal behavior, while problematic alcohol use played a moderating role in this relationship. These results highlight the pivotal influence of stress in the manifestation of suicidal behavior.

### Emotional regulation, suicidal behavior, and use of technology

Emotional regulation (ER) refers to “the processes by which individuals influence their emotions, when they have them, how they experience them and how they express them” ([59], p.275). It can occur either before (antecedent-focused) or after the generation of emotion (response-focused) [60] and it stands as a cornerstone in fostering optimal psychological well-being and serves as a crucial element in the mechanism of effective adaptation.

During stressful situations, emotional regulation plays an important factor for effective coping, since the situation requires individuals’ understanding their own thoughts, behaviors, emotional reactions, and social interactions [61]. Some studies have associated difficulties regulating emotions with suicidal ideation [58, 62,63]. Consistent with these results, several systematic reviews [64,65] concluded that ER may act as a protective factor for SR.

Difficulty with ER has also been associated with the development of PIU [53, 66, 67]. A study by Paulus et al. [68] suggested that lower levels of ER are involved in the development of GD, with individuals who manifest GD patterns showing a high risk of sadness, suicidal ideation, and suicidal plans. Individuals who have poor ER tend to use social media platforms with the aim of trying to self-regulate, resulting in dependence on them for some users [69]. Therefore, adaptive ER could be considered a protective factor against stressful situations and SR. Also, Arrivillaga et al. [39] suggested that PIU may be associated to SR, and that this association may be explained by trouble with impulse control, the overuse of internet as a coping mechanism, the access to information about suicide, and communication with groups or individuals that promote suicide.

### The present study

**Fig. 1:**
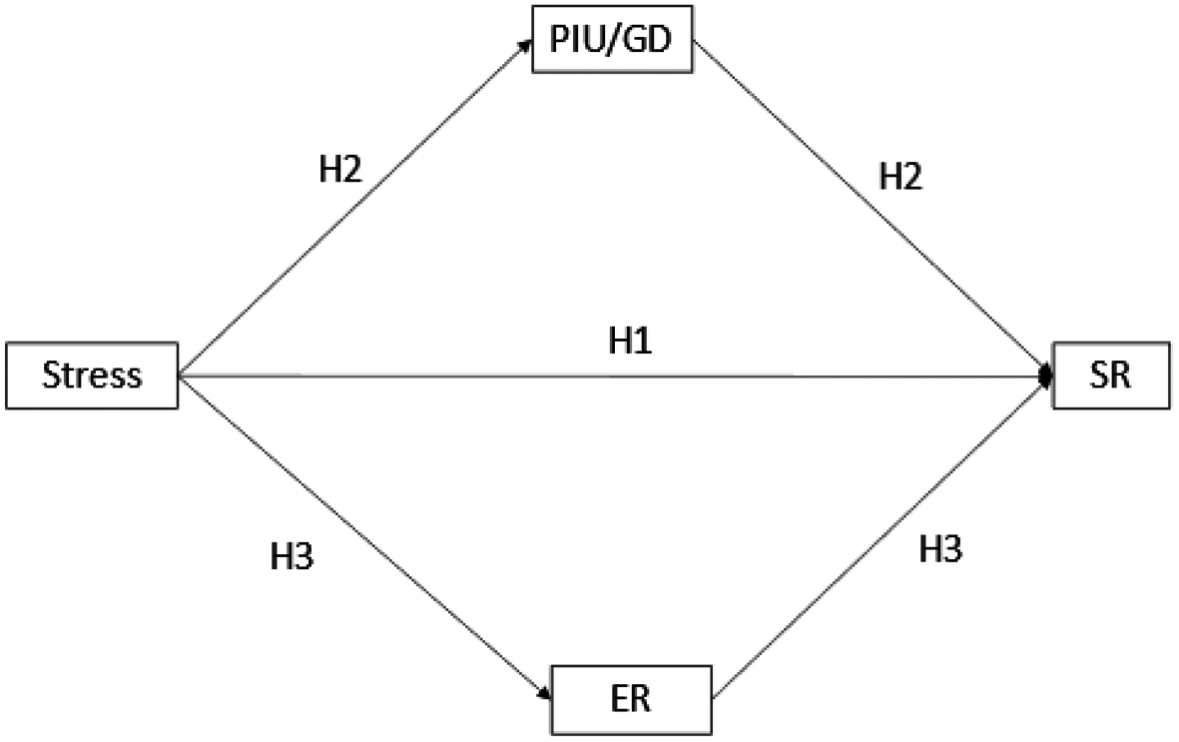
The mediation model used in the study. *Note*: PIU = Problematic internet use; GD = Gaming Disorder; SR = Suicidal risk; ER = Emotional regulation

As highlighted in the preceding section, various factors have been documented that seem to exert influence on SR The present study attempted to overcome the limitations of previous research and explored, as suggested by previous authors [9, 53] the relationship between SR, adolescent stress, difficulties in ER, and the intensive use of technology in a single design, The present study makes a significant contribution by addressing both the problematic use of technology in a general way (PIU), and specific uses of technology (GD) recognizing their potential harm and their associations with SR. Therefore, the aim of the present study was to examine whether stress, PIU/GD, and ER are associated with SR. Based on previous findings, it was hypothesized that: (i) high levels of stress would be associated with SR (H_1_), (ii) PIU/GD would mediate the relationships between stress and SR, that is, the effect of high stress on SR would be more intense among adolescents who exhibit PIU or GD (H_2_), and (iii) ER would mediate the relationship between stress and SR, that is, adolescents with low levels of ER would tend to show higher SR (H_3_) (see Fig. 1).

## Materials and methods

### Participants

A convenience sample of 430 adolescents completed an online survey. Over half the participants were female (58.3%), 39.5% male, and 2% defined themselves as non-binary. The mean age was 16.49 years (SD = 0.68), ranging between 16 and 19 years.

#### Measures

An ad hoc online survey was developed, containing several validated instruments. The survey included a *Sociodemographic and internet use questionnaire* for information regarding gender (female, male and non-binary), age, and number of weekly hours spent using the internet and playing videogames, as well as the following scales:

Perceived stress was evaluated using the abbreviated version of the *Perceived Stress Scale* (PSS-10; [70]; Spanish version: [71]). The PSS-10 comprises ten items that assess levels of perceived stress over the preceding month. Each item (e.g., *In the last month, how often have you felt nervous or stressed?*) is rated from 0 (*never*) to 4 (*very often*). Total scores can range from 0 to 40 and higher scores indicate higher levels of perceived stress. Items 6, 7, 8 and 9 are reversed. The PSS-10 showed acceptable internal consistency in the present study (α = 0.86).

Gaming disorder was evaluated using the *Internet Gaming Disorder Scale-Short Form* (IGDS9-SF; [72]; Spanish version: [73]). This scale comprises nine questions where the severity of GD and its effects are evaluated, both in the use of online and offline games in a 12-month period. Each item (e.g., *Have you continued gambling despite knowing that it was causing you problems with other people (partner, friend or family)?*) is rated on a five-point Likert scale, ranging from 1 (*Never*) to 5 (*Very often*). Total scores can range from 9 to 45 and higher scores indicate greater risk of IGD. The IGDS9-SF showed acceptable internal consistency in the present study (α = 0.80).

Problematic mobile phone use was evaluated using the *Mobile Related Experiences Questionnaire* (CERM; [74]). The scale comprises 10 items that assess mobile phone abuse and the problems caused by its emotional and communicative use. Each item (e.g., *Do you think that life without a mobile phone is boring, empty, and sad?*) is rated on a four-point Likert scale that goes from 1 (*Almost never*) to 4 (*Almost always*). Total scores can range from 10 to 40 and higher scores indicate higher levels of problematic mobile phone use. The CERM showed acceptable internal consistency in the present study (α = 0.80).

Emotion regulation was evaluated using the *Trait Meta-Mood Repair Scale* (TMMS-24; [75]; Spanish version: [76]). The scale comprises eight items that assess the ability to regulate emotions appropriately. Each item (e.g., *I try to have positive thoughts, even if I feel bad*) is rated on a five-point Likert scale ranging from 1 (*not at all agree*) to 5 (*strongly agree*). Total scores can range from 8 to 40. Scores less than 23 indicate the need to improve regulation, scores between 24 and 35 indicate adequate regulation, and scores above 36 indicate excellent regulation. The TMMS-24 showed acceptable internal consistency in the present study (α = 0.84).

Suicidal behavior was assessed using the *Revised Suicidal Behaviors Questionnaire* (SBQ-R; [77]; Spanish version: [28]). The scale comprises four items designed to identify lifetime suicidal ideation and attempts, suicidal ideation and suicide attempts in the past 12 months, communication of suicidal intent, and likelihood of a future suicide attempt among adolescents and young adults [28, 78]. The first item is scored using a Likert scale: from 1 [*never*] to 4a [*I tried to kill myself, but I did not want to die*] and 4 b [(*I* tried to kill myself, and I really expected to die]. Item 2 is scored on a scale ranging from 1 [*never*] to 5 [*very often*]. Item 3 is scored on a scale from 1 [*no*] to 3a [*Yes, more than once, but I really did not want to dieYes, more than once, but I really did not want to die*] and 3 b [*Yes, more than once, and I really wanted to do it*]. The fourth item is scored on a 7-point Likert scale: from 0 [*never*] to 6 [*very likely*].,Total scores from 3 to 18, where higher scores reflect an increased risk of suicidal behavior. Scores > 7 in the general population indicate a high risk of suicide. The SBQ-R showed acceptable internal consistency in the present study (α = 0.80).

### Procedure

The study used an online survey administered through the *Google Forms* platform and recruited participants from December 2021 to March 2022. The survey was completed by groups of Spanish baccalaureate students during tutoring hours so that they could respond via mobile phone, tablet, or computer. Participants received information regarding voluntary participation, confidentiality, and anonymity, which was provided at the beginning of the survey, and participants had to give their prior consent to participate in the study by pressing a ‘Yes/No’ button prior to responding. Once the data were collected (and due to its sensitivity), they were stored securely on the university server of the principal investigator (PI). All data were deidentified and only the PI had access to it, to guarantee its confidentiality and security [79]. To access the data, specific permission was requested from the PI, requiring those strictly necessary for subsequent analysis. The present study was approved by the Commission of Ethics in Animal and Human Experimentation (CEEAH) of the Autonomous University of Barcelona (ref. number CEEAH 3850). To ensure ethical considerations, a voluntary contact option was incorporated at the end of the survey. Participants were given the opportunity to indicate if any health-related issues were detected and if they wished to be notified. If so, participants were asked to provide their email address. Those adolescents who showed scores suggestive of suicidal risk were sent an informational email to inform and encourage them to talk to their parents, family doctor or get a local mental health referral.

#### Data analysis

The study was carried out utilizing a cross-sectional design. The data were analyzed with version 17 of the Stata program for Windows. Descriptive statistics and correlations between variables were calculated. Differences in the variables based on gender were calculated. A path analysis, with maximum likelihood estimation, was used to test the hypothesized model. Conventional criteria were used to assess the fit of the hypothesized model to the observed data. Adequate fit was inferred when TLI and CFI > 0.95, RMSEA < 0.06, SRMR < 0.08 [80, 81].

## Results

### Prevalence of suicide behavior risk

Among the total sample, 34.2% of the adolescents (*N* = 147) were at risk for SB (following the criterion established in SBQ-R scale: total score ≥ 7). Results also indicated that 30.7% had experienced suicidal ideation at some point in their life, 12.1% had at least one plan to die by suicide, and 5.1% had attempted suicide. Moreover, 22.1% had communicated to other individuals that they had thought about dying by suicide (*N* = 95). Finally, the probability of SB in the future was only reported by three participants (0.7%).

### Stress, suicide behavior, PIU/GD, and emotional regulation: gender differences

Statistically significant differences based on gender were observed for all variables (see Table 1). The post-hoc analysis showed that compared to females, males reported higher levels of emotional regulation (mean difference = 1.79; *p* <.014), and GD (mean difference = 3.94; *p* <.001), and lower levels of perceived stress (mean difference = -5.09; *p* <.001) and PIU (mean difference= -1.80; *p* <.001). Gender non-binary individuals reported higher levels of SR than males (mean difference = 6.05; *p* <.001) and females (mean difference = 5.06; *p* <.001).

Females reported higher levels of weekly hours spent on social networking sites than males (mean difference = 6.34; *p* <.05). Finally, non-binary individuals reported higher levels of weekly hours pent gamingthan both males (mean difference = 19.93; *p* <.001) and females (mean difference = 30.61; *p* <.05).

**Table 1:** ANOVA results for study variables

Gender	Female	Male	Non-binary	F (Sig.)
Age	16.52 (0.71)	16.46 (0.61)	16.28 (0.68)	0.856
ER	22.77 (6.25)	24.56 (5.93)	21.67 (6.50)	4.71*
Stress	23.22 (5.85)	18.13 (6.23)	21.44 (6.33)	36.39**
GD	12.45 (4.77)	16.40 (5.28)	16.00 (6.38)	31.97**
PIU	19.89 (4.38)	18.09 (3.91)	20.89 (7.15)	9.75**
SR	6.83 (3.24)	5.83 (2.62)	11.89 (5.35)	19.33**
Social networking	34.51 (22.51)	28.17 (22.46)	41.22 (50.14)	4.44*
Gaming	9.10 (11.81)	19.78 (19.22)	39.71 (87.13)	23.84**

ER = Emotional regulation; GD = Gaming disorder; PIU = Problematicinternet use; SR = Suicidal risk; Social networking = Hours spent using online social networks; Gaming = Hours spent playing videogames

*Note*: *p <.05; **p <.001

### Associations between stress, suicide risk, PIU/GD, and emotion regulation: correlation analysis

The results of the correlation analysis (Table 2) showed positive correlations that were statistically significant between stress, PIU and SR, and negative correlations between emotional regulation, stress, PIU, and SR. Age only weakly correlated with stress.

**Table 2:** Pearson’s correlations matrix for study variables

Variable	M	SD	1	2	3	4	5	6	7	8
1. Age	16.49	0.68	1	− 0.092	0.11*	0.000	0.026	0.057	0.005	− 0.100*
2. ER	23.43	6.18		1	− 0.255**	− 0.039	− 0.176**	− 0.283**	− 0.129**	0.024
3. Stress	21.17	6.48			1	− 0.027	0.286**	0.308**	0.088	− 0.197**
4. GD	14.08	5.36				1	0.241**	0.174**	− 0.008	0.273**
5. PIU	19.20	4.25					1	0.151**	0.291**	0.034
6. SR	6.54	3.19						1	0.127**	0.101*
7. Social networking	32.14	23.50							1	0.482**
8. Gaming	13.96	20.24								1

ER = Emotional regulation; GD = Gaming disorder; PIU = Problematic internet use; SR = Suicidal risk; Social networking = Hours spent using online social networks; Gaming = Hours spent playing videogames

*Note*: *p <.05; **p <.001

#### The mediation model

**Fig. 2:**
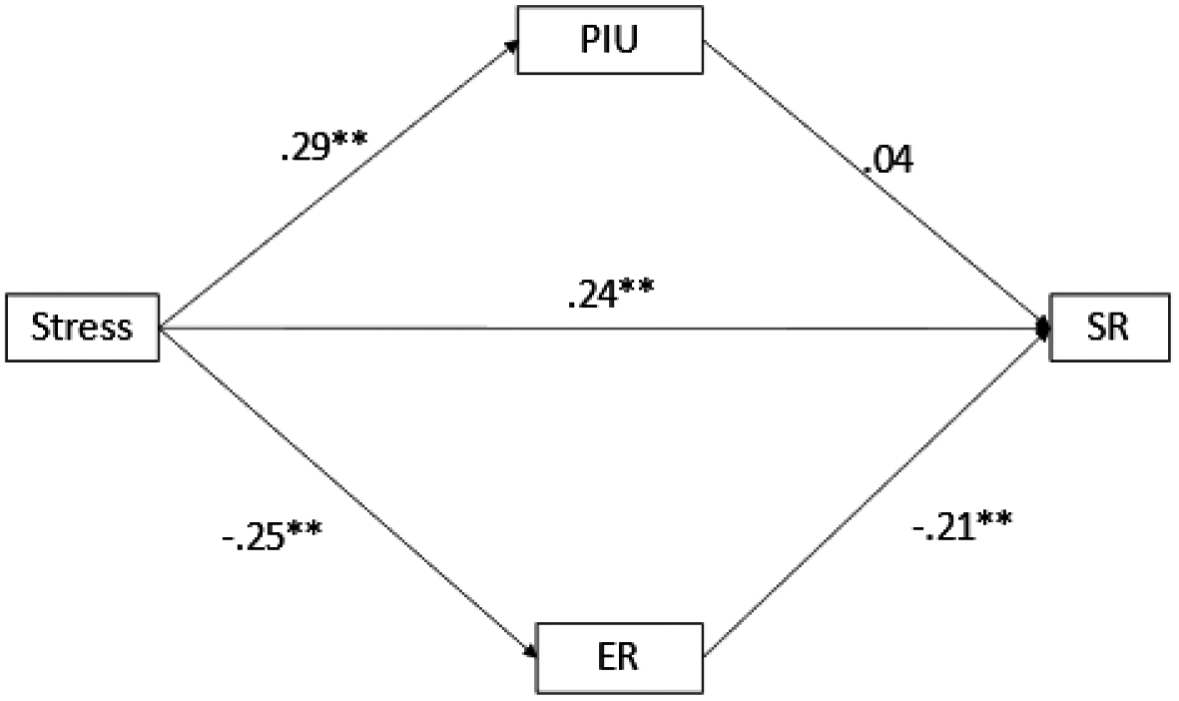
Results of the mediation analysis for PIU and ER as mediators. *Note*: PIU = Problematic internet use; GD = Gaming Disorder; SR = Suicidal risk; ER = Emotional regulation

**Fig. 3:**
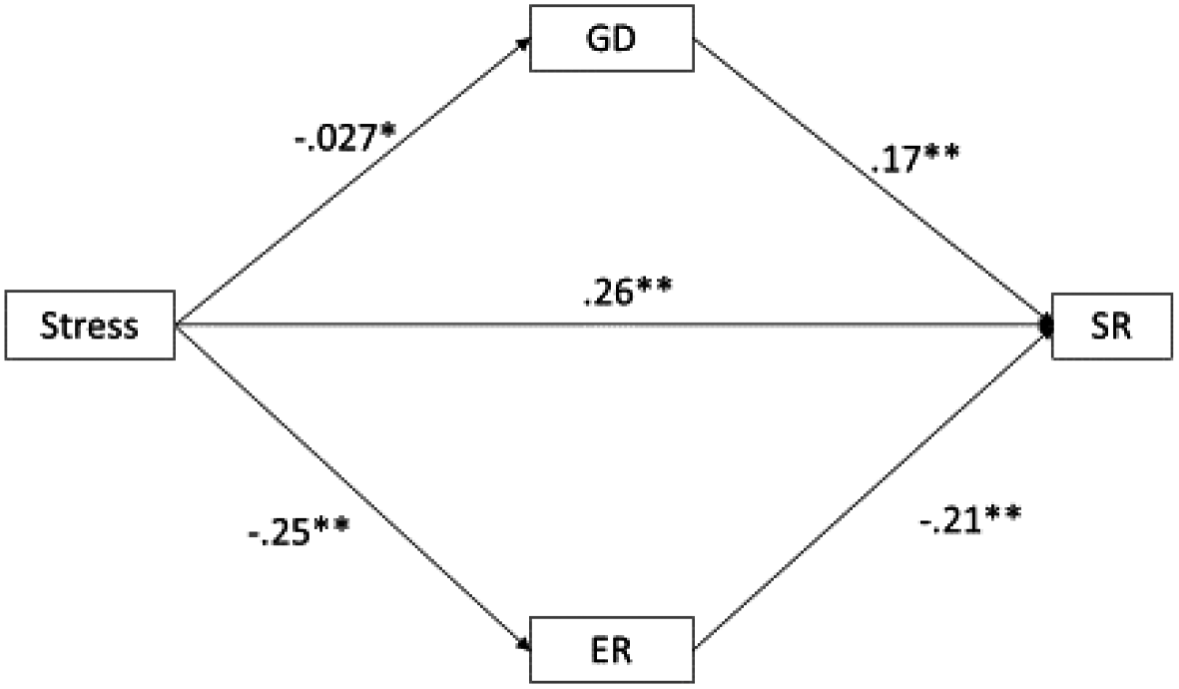
Results of the mediation analysis for GD and ER as mediators. *Note*: PIU = Problematic internet use; GD = Gaming Disorder; SR = Suicidal risk; ER = Emotional regulation

The results of the mediation model are presented in Figs. 2 and 3. As can be seen in Fig. 2, a positive association was found between stress and SR (β = 0.24; *p* <.001), and stress and PIU (β = 0.29; *p* <.001), and a negative association was found between stress and emotional regulation (β =-0.25; *p* <.001). Emotional regulation showed a negative association with SR (β =-0.21; *p* <.001) but PIU did not (β = 0.04; *p* >.05). The fit of the model was acceptable (CFI = 0.966; RMESEA = 0.10; SRMR = 0.033). The model explained 18.4% of the variance of SR. When PIU was replaced by GD (see Fig. 3), the direct relationship between stress and SR (β = 0.26; *p* <.001), and stress and ER (β=-0.25; *p* <.001) was maintained, as well as with GD (β=-0.027; *p* <.05). Emotional regulation was negatively associated with SR (β=-0.21; *p* <.001) and GD was positively associated with SR (β = 0.17; *p* <.001). The fit of the model was acceptable (CFI = 1; RMESEA = 0; SRMR = 0.015). The model explained 12.6% of the variance of SR.

## Discussion

The objectives of the present study were to examine whether stress, PIU/GD, and ER were associated with SR. It was hypothesized that stress would have a direct effect on SR, and that it would also be mediated by PIU/GD and ER. However, the first notable result in the study was the high prevalence of SR, according to Osman’s et al. criteria [77]. Previous studies that used the same scale among Spanish samples reported similar prevalence (34,7%) among adolescents [58]. The results corroborate the suggestions of some authors (e.g., [11]), that currently, after the COVID-19 pandemic, there was an increase in SR, especially among young populations.

 ThisThe results obtained in the present study partially support the proposed hypotheses. More specifically, the first hypothesis was confirmed. Stress appeared to be a risk factor for SR, as previous studies have suggested [55, 78, 82]. This may be because adolescents are going through a critical period of development with major biological, social, and psychological changes, and they do not have sufficient resources to cope with the situation showing difficulties in adapting to these changes or other daily stressors (e.g., COVID-19 pandemic, technological changes). This corroborates what various suicide theories have already suggested– that stress can be understood as an important factor influencing SR [45].

Regarding the second hypothesis, the results partially support the positive relationship between stress and SR mediated by the PIU/GD. The literature has suggested that stress plays an important role in the development of PIU [83]. This is because individuals try to cope with stress through easily accessible behaviors, including the use of various applications on smartphones and computer devices [48], which contribute to an increased risk of PIU [47]. The absence of a relationship between PIU and SR contradicts what was suggested in previous studies, both in the Spanish population [40, 43], and in other studies in different cultures [36, 84]. The results of the present study contradict the idea that PIU could be a suicide risk factor [11, 85]. Colmenero-Navarrete et al. [40]. had already noted the confusion concerning the relationship between PIU and SR, given that the intensive use of online social media platforms can be associated with negative consequences (e.g., cyberbullying, access to information on methods of suicidal behavior), and on the positive side it can be a method to obtain emotional support from others online. A possible explanation is that studies have been conducted with scales not specifically designed to address suicidal behavior [11]. Also, other factors associated to PIU, such as the presence of anxiety or depression, isolation, and use of the internet as an escape coping strategy, may be mediating this relationship [11]. These results provide support for previous authors' findings [40] suggesting that the effect of internet use on suicidality is unclear. However, and considering that internet may be a way to access to websites with pro-suicide information, as well as facilitation of suicide pacts and suicidal methods, PIU should be considered as a risk factor for suicidal behavior [11].

The present study's results support the relationship between stress and GD. This is consistent with previous studies [16, 83], and may be because of the use of videogames to escape from reality (e.g., [86]) or, according to the I-PACE model, as a copying strategy when suffering from stress [87]. However, in the present study, GD appeared to pose a risk for SR. This relationship is consistent with previous findings in the literature, which suggest an effect on suicidal ideation and possibly on suicide attempts [9]. The relationship between GD and SR may be explained because the problematic gamer, who is an intensive gamer (e.g., [52]), may be exposed to elements associated to suicidality, such as distress [88], cyberbullying [89] or the co-occurrence of other common third variables (e.g., escapism, depression) [90–91]. However, to date, some issues remain in this relationship, because one study [92] suggested that GD may be a protective factor for suicidal ideation when the confounders were adjusted (9). The results of the present study also suggest that when assessing SR in a composite manner and GD based on the presence of addiction symptoms, the relationship is confirmed.

On the other hand, it was found that as ER increased, SR decreased. These results concur with previous studies [63, 65, 93]. The relationship between poor ER and SR is associated with the presence of stress. The present study’s results concur with previous ones which highlight that psychological stress is associated with ER [94] and with SR [43, 55, 82].

The results here support the idea that perceived stress is a risk factor for SR, especially among those adolescents with poor ER and GD. Moreover, and considering the cross-sectional nature of the present study's data, it cannot be ruled out that other mechanisms are involved in the development of SR. For instance,Arrivillaga et al. [43] suggested that the presence of depressive symptoms may mediate the relationships between stress and problematic use of social media. Therefore, it is possible that other mediating factors are implicated in the suggested relationships. Therefore, future studies with a greater number of variables, and controlling for potential confounders, are recommended. Also, studies exploring potential relationships in the reverse direction, or even bidirectional relationships are warranted.

Therefore, the development of skills to cope with stress, to better manage emotions and videogaming would likely improve psychological adjustment [95] and could prevent the negative effects of stress in facilitating suicidal behaviors from the early stages of adolescence [44].

### Limitations and future directions

While the present study contributes towards an understanding of the mechanisms underlying stress and suicide ideation by using a reasonably sized sample of adolescents, it has some limitations. The main shortcoming concerns the convenience sampling, the use of self-report data, and the cross-sectional design, which limit the generalization of the results and the determination of causal relationships between the study variables. Future studies should use longitudinal methodologies and more representative samples.

## Conclusions

The present study strengthens the evidence that perceived stress could become a risk factor for suicide among adolescents, especially among those with poor ER and GD. Considering the prevalence of suicide among adolescents and young adults, the results of the present study suggest that suicide prevention programs should include emotional regulation strategies, stress coping skills, and videogaming management skills in early stages of high school. Providing these protective resources to adolescents could help them face the stressful and challenging situations typical of adolescence and will help them have greater well-being and satisfaction with their life.

## Data Availability

The datasets used and/or analysed during the present study are available from the corresponding author on reasonable request.
